# Rhythmicity of Prefrontal Local Field Potentials after Nucleus Basalis Stimulation

**DOI:** 10.1523/ENEURO.0380-21.2022

**Published:** 2022-02-03

**Authors:** Balbir Singh, Xue-Lian Qi, David T. Blake, Christos Constantinidis

**Affiliations:** 1 Department of Biomedical Engineering, Vanderbilt University, Nashville, TN 37235; 2 Department of Neurobiology and Anatomy, Wake Forest School of Medicine, Winston-Salem, NC 27157; 3 Department of Neuroscience and Regenerative Medicine, Medical College of Georgia, Augusta University, Augusta, GA 30912; 4 Neuroscience Program, Vanderbilt University, Nashville, TN 37235; 5 Department of Ophthalmology and Visual Sciences, Vanderbilt University Medical Center, Nashville, TN 37232

**Keywords:** acetylcholine, basal forebrain, LFP, monkey, neurophysiology, working memory

## Abstract

The action of acetylcholine in the cortex is critical for cognitive functions and cholinergic drugs can improve functions such as attention and working memory. An alternative means of enhancing cholinergic neuromodulation in primates is the intermittent electrical stimulation of the cortical source of acetylcholine, the nucleus basalis (NB) of Meynert. NB stimulation generally increases firing rate of neurons in the prefrontal cortex, however its effects on single neurons are diverse and complex. We sought to understand how NB stimulation affects global measures of neural activity by recording and analyzing local field potentials (LFPs) in monkeys as they performed working memory tasks. NB stimulation primarily decreased power in the alpha frequency range during the delay interval of working memory tasks. The effect was consistent across variants of the task. No consistent modulation in the delay interval of the task was observed in the gamma frequency range, which has previously been implicated in the maintenance of working memory. Our results reveal global effects of cholinergic neuromodulation via deep brain stimulation, an emerging intervention for the improvement of cognitive function.

## Significance Statement

Stimulation of the endogenous source of acetylcholine in the cerebral cortex, the nucleus basalis (NB) of Meynert has shown promise as an alternative to cholinergic drugs, which are frontline medications for the treatment of Alzheimer’s disease and age-related dementia. The neural mechanisms through which stimulation may improve cognitive function are incompletely understood. Here, we investigate the effects of NB stimulation in the prefrontal cortex by examining the power of the local field potentials (LFPs) during execution of working memory tasks.

## Introduction

The forebrain cholinergic system is essential for the performance of cognitive functions (Sarter et al., 2016; Galvin et al., 2018). Cognitive decline in age-related dementia and Alzheimer’s disease occurs in parallel with cholinergic degeneration (Terry and Buccafusco, 2003; Ballinger et al., 2016). Animal models similarly exhibit profound cognitive deficits following cholinergic depletion (Chudasama et al., 2004; Croxson et al., 2011). Conversely, cholinergic agonists and cholinesterase inhibitors can improve cognitive function (Birks and Harvey, 2018), as can stimulation of the endogenous source of acetylcholine in the cerebral cortex, the nucleus basalis (NB) of Meynert (Mesulam et al., 1983). Recent experiments in non-human primates have demonstrated the feasibility of cholinergic deep brain stimulation in improving cognitive function, in young adult animals (Blake et al., 2017; Liu et al., 2017, 2018). Pilot studies have also been conducted in humans, with encouraging results in terms of the safety and tolerance of the procedure, although no dramatic cognitive improvements have been demonstrated so far (Freund et al., 2009; Kuhn et al., 2015; Hardenacke et al., 2016). Substantial obstacles remain for clinical translation of cholinergic deep brain stimulation, including that considerable degeneration of the basal forebrain may have already occurred by the time of a clinical dementia diagnosis (Nazmuddin et al., 2021).

Investigating the effects of NB stimulation on neuronal activity is essential for understanding its mechanism of action and optimizing its effectiveness (Subramaniam et al., 2021). NB stimulation affects neuronal activity in the prefrontal cortex during performance of working memory tasks (Qi et al., 2021). Reported results generally indicated an increase in mean firing rate of prefrontal neurons; however, the effects were considerably diverse, including a substantial minority of neurons being suppressed by stimulation. The effects of stimulation on measures of population activity and rhythmicity such as the local field potential (LFP) have not been known. We were motivated therefore to investigate effects of NB stimulation by examining the power of the prefrontal LFP.

LFP represents summation of ionic currents in a limited cortical volume in the order of 0.1- to 0.2-mm radius (Buzsaki, 2004; Kajikawa and Schroeder, 2011). During presentation of stimuli, correlated bottom-up inputs can serve to synchronize population neuronal spiking, and phases of synchronized excitation by pyramidal neurons followed by inhibition by interneurons can thus produce rhythmicity in the field potentials (Fries, 2009). LFP rhythmicity in the γ frequency range in particular is well known to emerge in the delay period of working memory tasks and to be tuned for information held in memory, e.g., spatial location of a remembered cue (Pesaran et al., 2002; Holmes et al., 2018). γ Oscillations are highly detectible in other extracellular field recordings (such as EEG or MEG) and are thus a reliable marker of underlying cognitive processes impacting neural circuit interactions (Uhlhaas and Singer, 2011; Roux and Uhlhaas, 2014). Our current study investigated the impact of NB stimulation on LFP potential measures of rhythmic firing during performance of working memory tasks.

## Materials and Methods

Two male, rhesus monkeys (*Macaca mulatta*) aged 12–13 years old, and weighing 7–10 kg were used in this study. All experimental procedures followed guidelines by the United States Public Health Service Policy on Humane Care and Use of Laboratory Animals and the National Research Council’s Guide for the Care and Use of Laboratory Animals and were reviewed and approved by the Institutional Animal Care and Use Committee (IACUC).

### Surgery and neurophysiology

**Figure 1.**
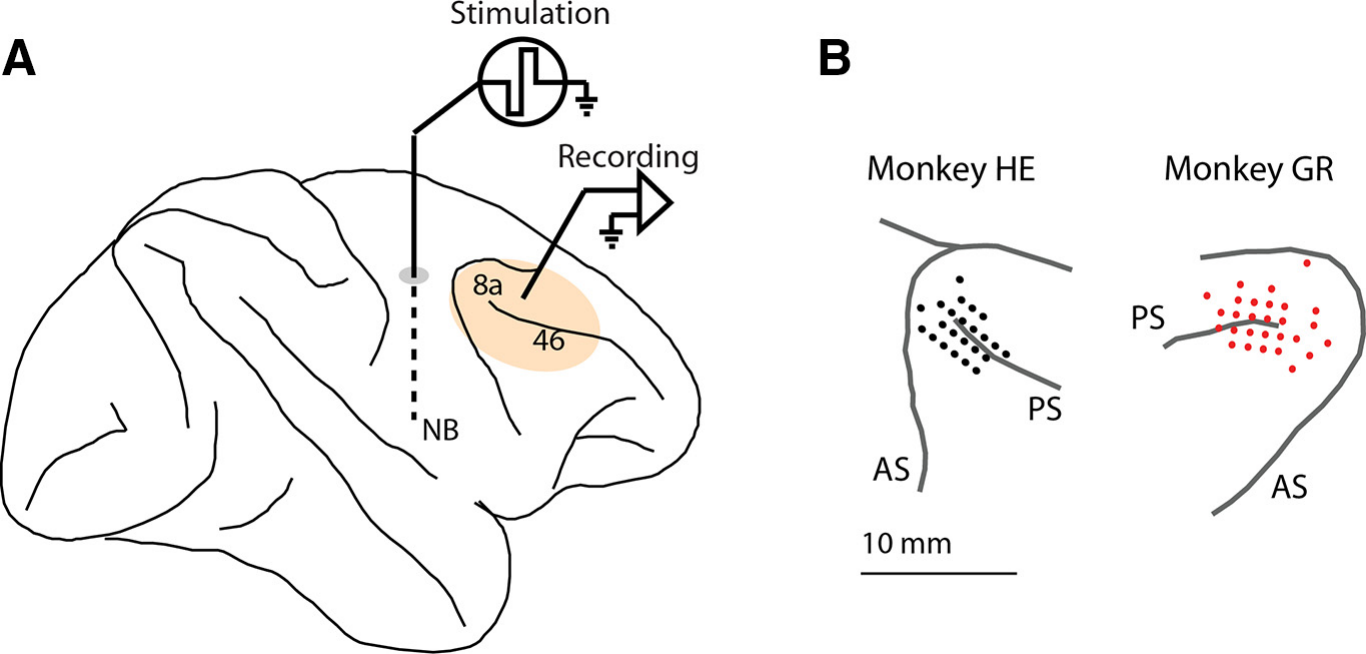
Stimulation and recording areas. ***A***, Schematic diagram of the monkey brain, with the cortical region sampled with neurophysiological recordings and relative location of the stimulation recording indicated. ***B***, Penetration maps of where neurons and LFPs were recorded in two animal subjects (HE and GR). PS, principal sulcus; AS, arcuate sulcus.

A 20 mm-diameter recording cylinder was implanted over areas 8a and 46 (Fig. 1*A*), residing in the posterior aspect of the dorsolateral prefrontal cortex (Riley et al., 2017). Recordings of single units and LFPs were obtained with arrays of epoxylite-coated tungsten microelectrodes with a 250-μm diameter and 1- to 4-MΩ impedance at 1 kHz (FHC). Typically, three to four electrodes were lowered into the cortex in a single recording session. The signal was split and differentially filtered via hardware means for single units (500 Hz to 8 kHz) and LFPs (0.5−100 Hz), and LFP data were captured at a 500-Hz sampling rate. Deep brain electrodes reaching the NB were implanted unilaterally (in the left, and in the right hemisphere, respectively for the two animals) at 8 mm lateral, 16 mm anterior interaural, and 29 mm below the cortical surface in a vertical penetration. Stimulation electrodes were fabricated in-house based on published specifications (McCairn and Turner, 2009). Conductors were 50 μm Pt/Ir, Teflon-insulated wire (AM Systems). The last 0.7 mm of insulation was stripped to achieve impedances of 5–10 kΩ at 1 kHz. Stimulation pulses were created by an isolated pulse stimulator (Model 2100, AM Systems), which was controlled by custom programed software, written on the MATLAB computational environment (MathWorks). Stimulation was delivered with biphasic, negative first, unipolar 200-μA pulses with 100 μs per phase, and 80-Hz pulses were delivered for 15 s.

### Behavioral tasks

**Figure 2.**
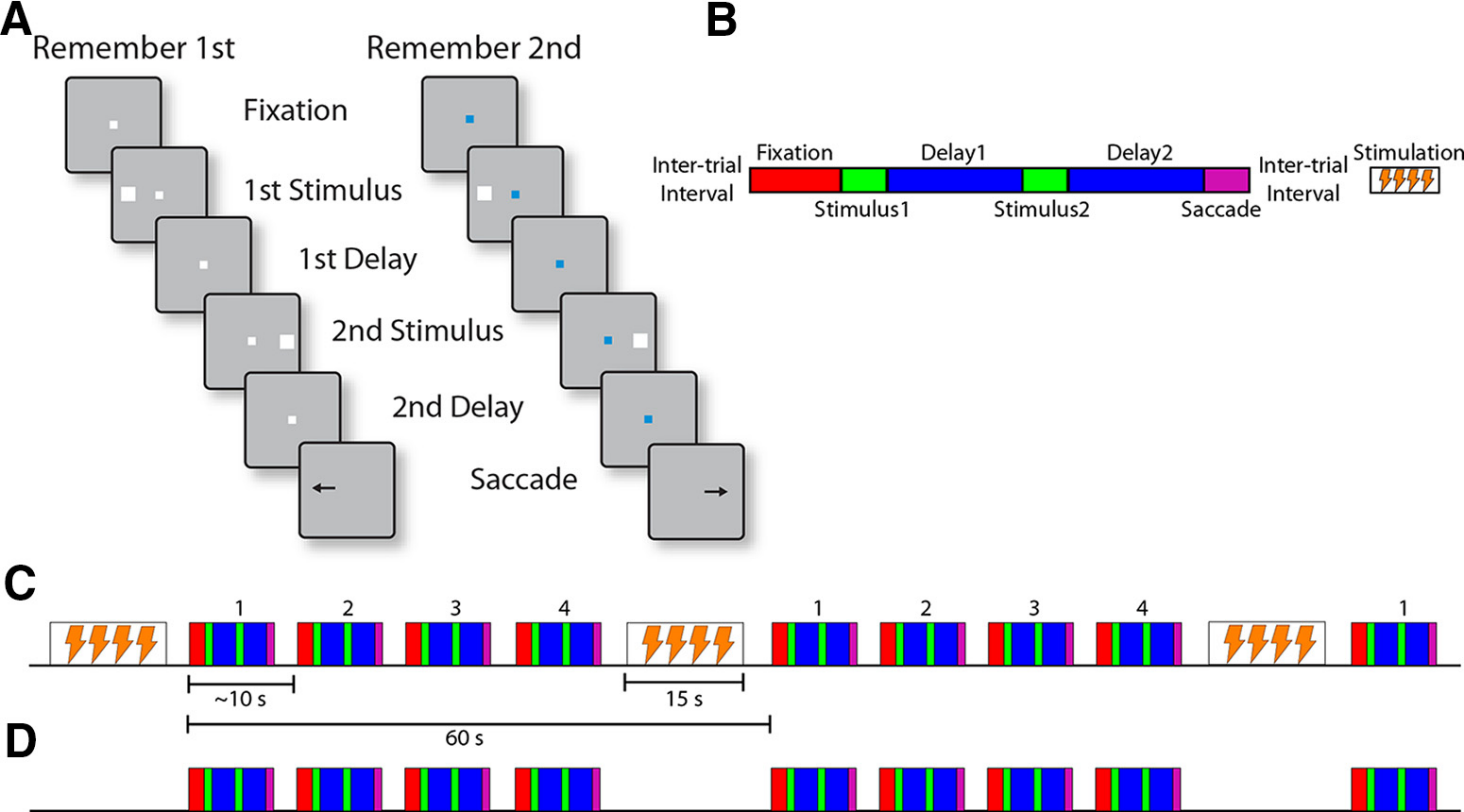
Behavioral task. ***A***, Successive frames illustrate the sequence of events in the behavioral task. Depending on the white or blue color of the fixation point, the monkey has to remember either the first or the second of two visual stimuli presented in sequence, respectively. At the end of the trial, the fixation point turns off and the monkey needs to perform an eye movement toward the remembered location of the visual stimulus. ***B***, Schematic diagram of a single trial of the task. Blocks represent the successive events in the task: fixation, first visual stimulus presentation, first delay period, second visual stimulus presentation, second delay period, and saccade. Successive trials are separated by intertrial intervals. NB stimulation, when delivered, always occurs during the intertrial interval. ***C***, Sequence of trials during a stimulation block, in a compressed time scale, relative to panel ***B***. Successive trials, labeled 1–4, each lasting ∼10 s, are followed by 15 s of stimulation. ***D***, Sequence of trials during a control (no-stimulation) block. The trials are arranged in the same fashion as in the stimulation block, including an extended intertrial interval every 60 s, during which however no stimulation is applied (sham).

The monkeys faced a computer monitor 60 cm away in a dark room with their head fixed. Eye position was sampled at 240 Hz, digitized, and recorded with an infrared eye position tracking system (model RK-716; ISCAN). The visual stimulus presentation and behavior monitoring were controlled by in-house software (Meyer and Constantinidis, 2005) implemented in MATLAB. The tasks used in the present study were variations of the oculomotor delayed response (ODR) task, but involving two visual stimuli appearing in sequence. The tasks required the monkey to remember and make an eye movement to the location of either the first or the second visual stimulus depending on the color of fixation point (Fig. 2*A*). After the animals fixated at a white/blue fixation point located at the center of the monitor for 1 s, two white squares (1.5° in size) were displayed sequentially for 0.5 s, with a 1-s intervening delay period. The first visual stimulus was displayed at one of eight possible locations arranged along a circular ring of 12° eccentricity, with a 45° angular separation between neighboring visual stimuli. The monkeys were trained with stimulus appearances at every possible location, however, during recordings the first stimulus appeared at one of these eight locations and its diametric location. We generally attempted to place one of the two stimuli in the receptive field of a neuron recorded from our electrodes, however recordings were performed with multiple electrodes and the stimulus may have appeared at any location of a neuron’s receptive field. This first stimulus followed by a delay period and then a second visual stimulus, which was displaced 0°, 45°, 90°, or 180° relative to the first. Two additional, “null” conditions were included, in which either the first or second stimulus presentation was omitted, so that there were 10 trial types in total, and these were used with equal frequency. After a second delay period of 1 s, the monkeys were required to saccade to the location of the first visual stimulus if the fixation point was white in color (remember-first condition), and to the location of the second visual stimulus if the fixation point was blue (remember-second condition). The monkeys were rewarded with juice after making a correct saccade. Deviating gaze beyond a 3° radius fixation window led to the immediate termination of the trial without reward. To minimize the uncertainty about the visual stimulus to be remembered, the remember-first and remember-second conditions were presented in blocks of trials. The animal was required to perform 10 correct trials of the remember-first task, before the task alternated to the remember-second condition. During sessions of NB stimulation that were delivered in block mode (trials with and without stimulation collected during the same daily session), ∼60 trials were collected for each block, which therefore involved six alterations between the remember-first and remember-second rule. A control block of trials was acquired first, followed by a stimulation block of trials. Intermittent stimulation was applied for 15 s at 80 pulses per second, followed by ∼45 s with no stimulation (Fig. 2*B*). Stimulation was applied in the inter-trial interval, after a trial had completed, and a new trial began after stimulation had elapsed.

### LFP analysis

We used the FieldTrip toolbox (Oostenveld et al., 2011) for preprocessing analysis and Chronux package (Bokil et al., 2010) for time-frequency analysis. For power analysis of LFP signals from the recording electrodes, a bandpass filter (0.5–100 Hz) was used. We removed line power (60 Hz) and other artifacts from each electrode and trial in the LFP signal, if present. We then used a multi-taper method to perform a power spectrum analysis of LFP. Time-resolved plots of power (spectrograms) were constructed and plotted after subtracting the mean power of the baseline period at each frequency. The baseline period was defined to be last 1 s in the inter-trial interval apart from the trials in which the NB stimulation was delivered (time >5 s after the onset of the fixation point). This occurred at least 1 s after the end of the trial and delivery of reward, at a time when the monkey was free to make eye movements, before the appearance of the next stimulus. Alternatively, we used the 1 s of fixation before the cue presentation as baseline for some analysis. We then compared the LFP power at each frequency between the control and simulation conditions. We also analyzed the LFP power at different frequency bands defined as α (8–14 Hz), β (20–35 Hz), and γ (45–100 Hz) after subtracting the mean power. We obtained estimates of the variability of these measures by using a bootstrap technique. We randomly selected with replacement 75% of trials from each of electrode and repeated the power spectrum estimation 1000 times. We compared power between different conditions (control vs NB stimulation; remember-first vs remember-second; correct vs error trials) on a time point by point fashion, averaging power over a 250-ms window, with a step of 100 ms. We then used a permutation test that randomized the label of trials across conditions while preserving information about which electrode each trial originated. This was evaluated at the α = 0.01 significance level with a two-tailed test.

## Results

Two monkeys were implanted with unilateral electrodes targeting the NB to determine the effects of stimulation on behavioral performance and neural activity. Electrode placement was guided by MR imaging (Fig. 1*A*). Neurophysiological recordings were then obtained from areas 8a and 46 of the dorsolateral prefrontal cortex (Fig. 1*B*). The monkeys performed a task requiring them to view two stimuli presented in sequence and make an eye movement to the remembered location of either the first or second of them (Fig. 2*A*). A white fixation point instructed the monkeys to remember the first stimulus; a blue fixation point required them to remember the second stimulus. The two stimuli could appear at any of eight possible locations, presented in a randomized order. When NB stimulation was delivered, it was applied during an inter-trial interval between two trials for 15 s, at a frequency of 80 pulses per second (Fig. 2*B*,*C*). The monkeys performed consecutive trials for 45 s (typically four to five completed trials), without NB stimulation. At the end of the trial that exceed the 45-s threshold, NB stimulation was applied, and the cycle was repeated. Blocks of trials were obtained first under control conditions and then under NB stimulation, in daily sessions.

### Effects of NB stimulation on LFP spectral power

We recorded LFPs from electrodes in the prefrontal cortex (areas 8a and 46) from which single-unit recordings were also obtained. The resulting dataset consisted of 7846 trials from 126 LFP sites in control condition and 6201 trials from 132 LFP sites in NB stimulation condition. Synchronization of LFP power in the γ frequency band has been proposed as the critical neural correlate of working memory maintenance (Lundqvist et al., 2018). We therefore examined the LFPs recorded from the prefrontal cortex, in the α (8–14), β (20–35), and γ (45–100) frequency bands, as these bands were defined in previous studies supporting the latter theory (Lundqvist et al., 2016), and tested whether the effects of NB stimulation increased γ power during the delay periods of the task.

**Figure 3.**
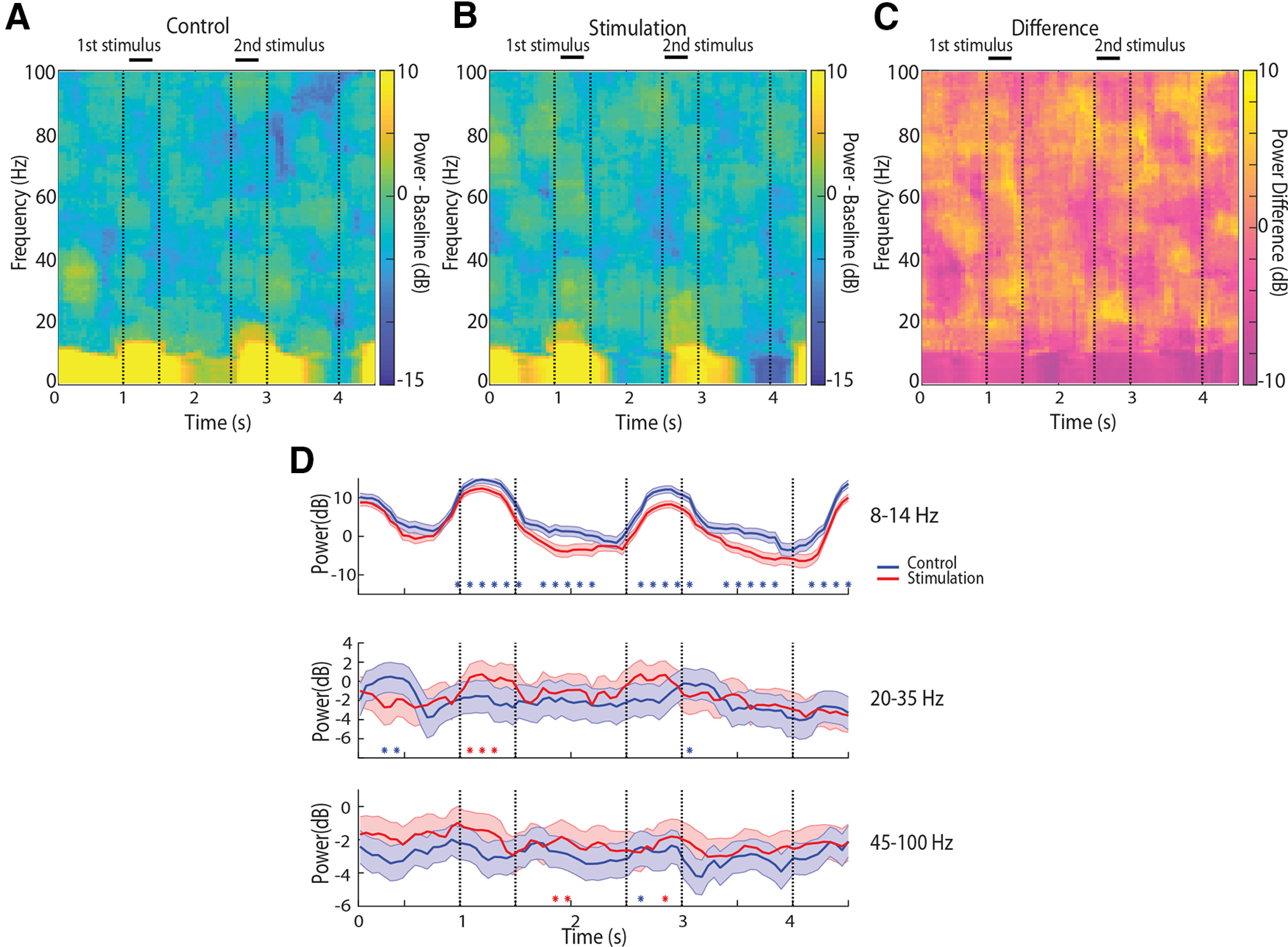
Time-resolved power of LFPs. ***A***, Spectrograms of LFPs recorded from the prefrontal cortex during the control condition, after subtracting the mean power of the baseline period at each frequency band (*n* = 7846 trials for the control and 6201 for the stimulation condition). Horizontal lines indicate time of stimulus presentations. ***B***, Mean power after subtracting the baseline during the stimulation condition. ***C***, Difference in power between stimulation and control. Positive values indicate higher power in stimulation condition. ***D***, Time course of spectral power, after subtracting the baseline, in the α, β, and γ frequency bands for the control and stimulation conditions. Shaded regions around each line represent 95% confidence intervals, estimated with a bootstrap method.

We calculated average spectral power during the course of the trial, after subtracting the mean power from a baseline period in the intertrial period. Data analyzed in this fashion allowed us to determine spectral power changes driven by task events in each trial and how these differed between conditions (shown in Fig. 3*A*,*B*). This analysis averaged all conditions, task types, and stimulus locations, including catch trials. The caveat in this analysis is that changes between stimulation and control condition that may be present after the end of the trial and delivery of reward may not be detectable, and we therefore omit comparisons at the end of the trial. The most salient effect of NB stimulation plotted was a decrease in the α frequency range (Fig. 3*C*,*D*). The effect was most pronounced during the stimulus presentations and the first delay period. We compared power in the same electrodes, and the same behavioral sessions, when NB stimulation was applied and when it was not. Stars in Figure 3*D* indicate individual time points where the difference statistical significance based on a permutation test evaluated at the 0.01 significance level.

**Figure 4.**
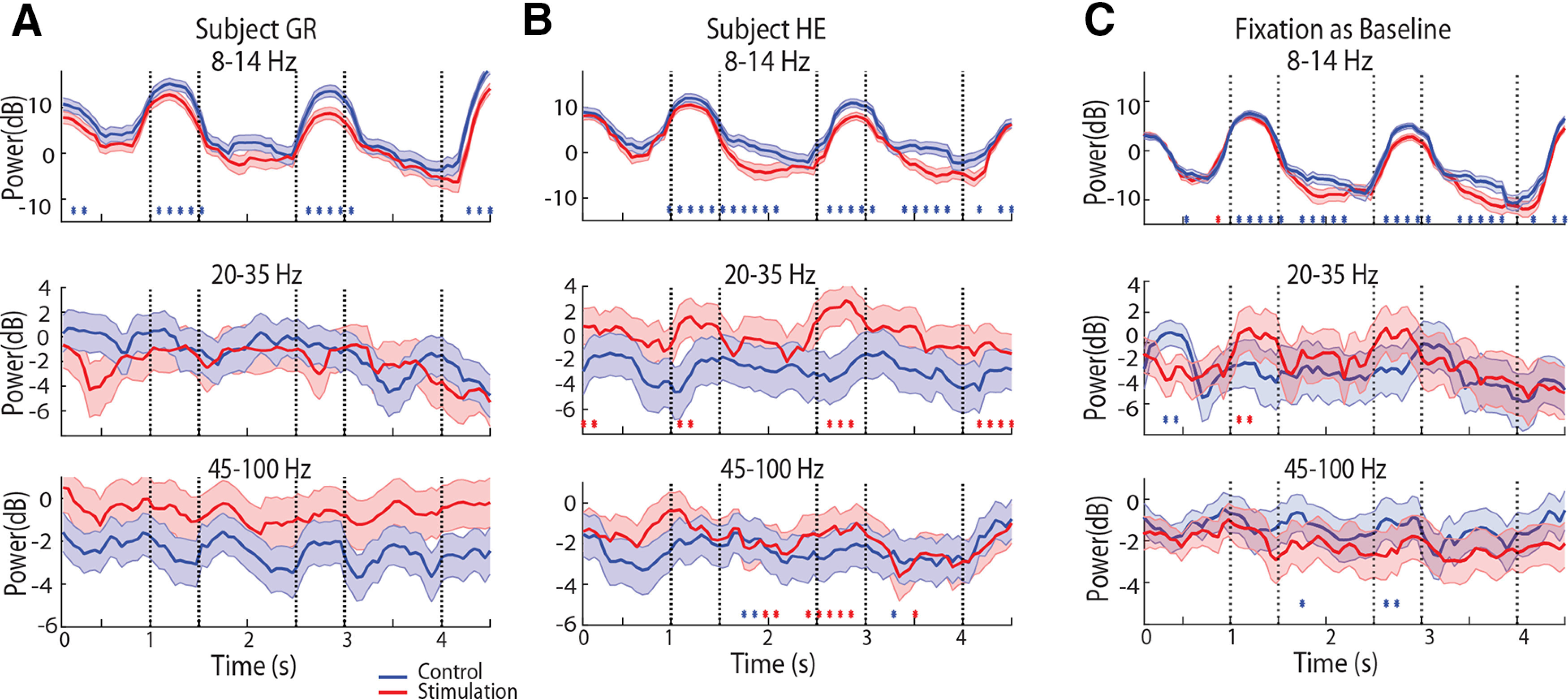
Spectral power for different subjects and baseline conditions. ***A***, Time course of spectral power, after subtracting the baseline, in the α, β, and γ frequency bands for the control and stimulation conditions plotting results from subject GR only. ***B***, As in ***A***, plotting results from subject HE only. ***C***, Time course of spectral power, after subtracting the fixation period as baseline. Stars represent significant difference between the control and NB stimulation conditions, evaluated with a permutation test, at the 0.01 significance level.

We also relied on a general linear model analysis, computing spectral power across each task epoch, in each trial, and comparing this power between epochs, and stimulation conditions. This analysis confirmed a highly significant main effect of NB stimulation (two-way ANOVA, *F*_(1,70225)_ = 335.7, *p* = 3.7 × 10^−79^). α Power computed in this was significantly lower under NB stimulation than control for each of the task epoch conditions (Tukey’s *post hoc* test, *p* < 0.05 for each of the fixation, stimulus1, delay1, stimulus2, delay2 periods). This decrease in α power was also evident in both subjects, when processed separately (Fig. 4).

NB stimulation produced more subtle effects in the β frequency band. β Power appeared elevated during the stimulus presentation periods, with several time points in that interval indicating a significant difference between conditions (Fig. 3*D*). However, the effect was driven by a single subject (Fig. 2*F*); for the second subject, the opposite trend of decreased β power with NB stimulation was evident. Averaging power across each task epoch revealed no significant difference between power in the control and NB stimulation condition in the β frequency band (two-way ANOVA, *F*_(1,70225)_ = 0.32, *p* = 0.57 for main effect of NB stimulation). No individual task epoch exhibited a significant difference in β power either (Tukey’s *post hoc* test, *p* > 0.05 for each of the fixation, stimulus1, delay1, stimulus2, and delay2 periods).

An increase in γ band power was also evident with NB stimulation. Individual time points reached significance in the delay and second stimulus period (Fig. 3*D*). Averaging power across each task epoch revealed a significant increase of γ power in the NB stimulation condition (two-way ANOVA, *F*_(1,70225)_ = 73.37, *p* = 1.09 × 10^−17^). This difference reached statistical significance in the fixation, stimulus1 and delay1 epochs (Tukey’s *post hoc* test, p < 0.05).

An important consideration in this analysis was the choice of baseline. We relied on the inter-trial interval, a period over which no stimuli were presented and animals were unconstrained to generate eye movements, which could potentially skew the results. We therefore repeated the analysis using the 1 s of the fixation period as baseline, instead (Fig. 4*C*). Spectral power relative to the fixation period produced a near identical pattern of significant differences between control and NB stimulation conditions in the α band (Fig. 4*C*, top). No systematic differences between conditions were present in the β frequency band (Fig. 4*C*, middle). γ Band differences were most affected by this choice of baseline, however NB stimulation produced even lower γ band power during the delay period (Fig. 4*C*, bottom). In other words, NB stimulation increases γ power from the fixation period onward, rather than specifically for the delay period of the task, so that using the fixation period as a baseline makes no increase in γ power apparent during the course of the trial.

**Figure 5.**
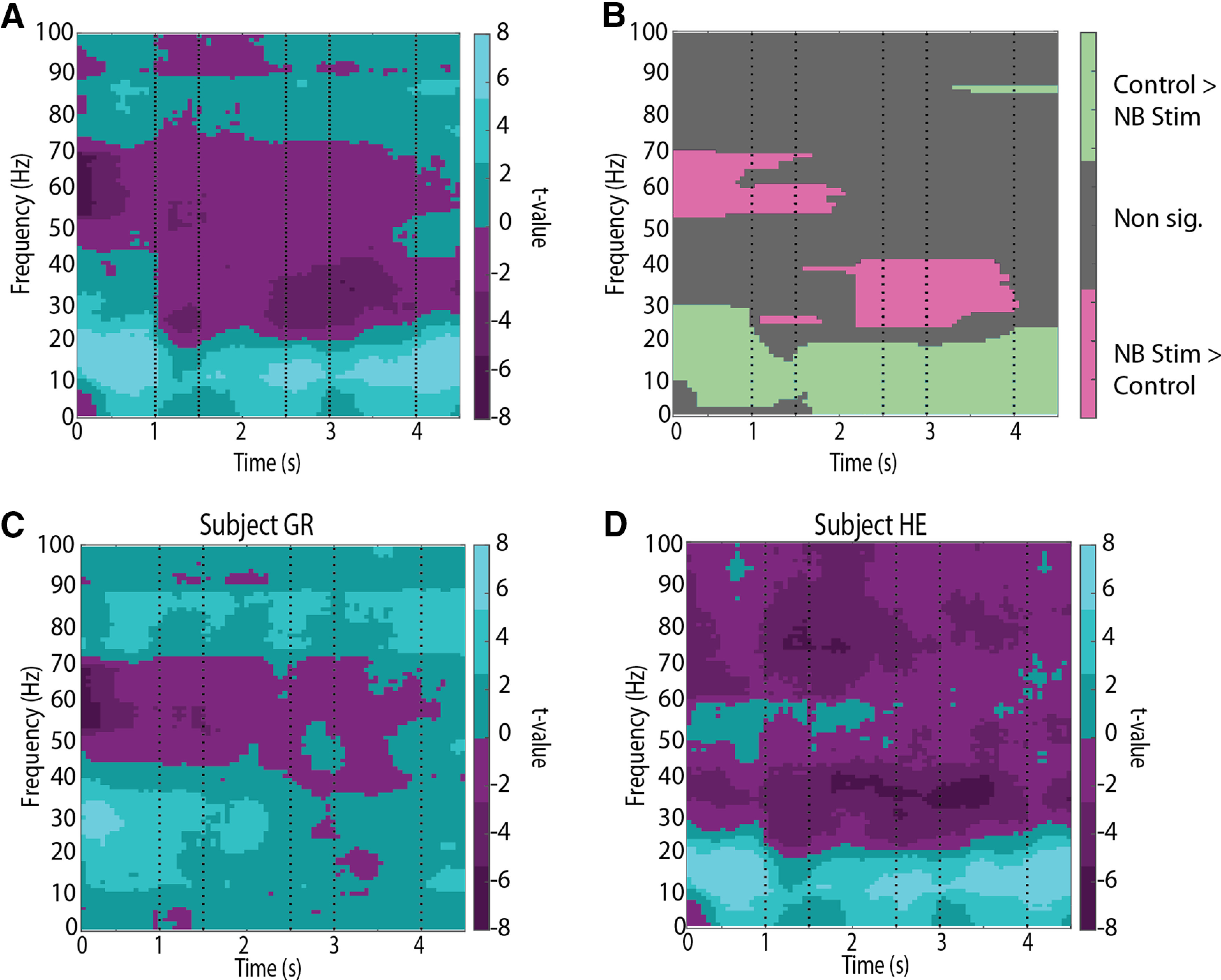
Cluster-based spectral analysis. ***A***, Color plot represents two-sample *t* test *t* values comparing the time point-by-time point LFP spectral power in the NB stimulation relative to the control condition. ***B***, Clusters of power difference between the control and NB stimulation condition, based on a permutation test that was based on *t* values shown in ***A***. Color plot at each point cluster represents the bootstrapped *p* value. ***C***, As in ***A***, plotting results from subject GR only. ***D***, As in ***A***, plotting results from subject HE only.

Another important consideration for our analysis was the choice of spectral bands. It is possible that our a priori selection of the three frequency bands where we averaged spectral power did not correspond well to frequency zones that were modulated by task factors. For this reason, we repeated our statistical analysis across the whole time-frequency space and identified clusters with a permutation-based approach (Maris and Oostenveld, 2007). The results are shown in Figure 5. The analysis revealed that our definition of the α band (8–14 Hz) corresponded well with a zone of spectral power that was reduced by NB stimulation between 8 and 18 Hz (Fig. 5*A*,*B*), which additionally did not overlap with our definition of β band. Similarly, a spectral zone of enhanced NB stimulation was observed between 25 and 40 Hz, which mostly coincided with our β band definition (20–35 Hz). No consistent effect was observed in the γ frequency band. Performing this analysis separately in the two animals confirmed our conclusions that α power modulation was most consistent between the two animals; effects in the β and γ frequency moved essentially in opposite directions (Fig. 5*C*,*D*).

### LFP spectral power across tasks

**Figure 6.**
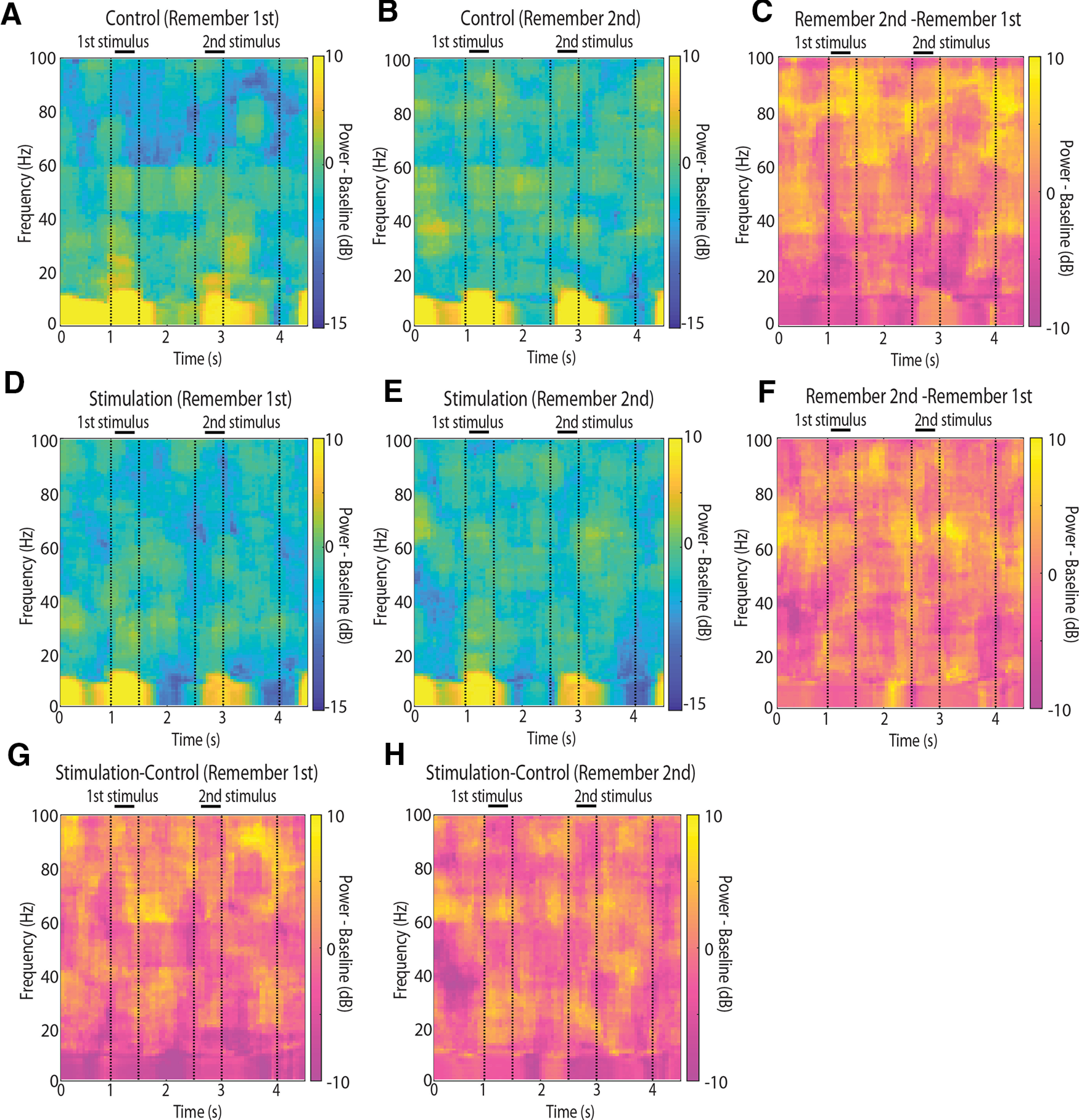
Power spectrum for remember-first and remember-second task. ***A***, Spectral power of LFPs recorded from the prefrontal cortex during the control condition in the remember-first task, after subtracting the mean power of the baseline fixation period at each frequency band (*n* = 3961 trials). ***B***, As in ***A***, for the remember-second task (*n* = 3885 trials). ***C***, Difference in power between remember-first and remember-second tasks in the control condition. ***D***, As in a ***A***, for the NB stimulation condition (*n* = 3143 trials). ***E***, As in ***B***, for the NB stimulation condition (*n* = 3058 trials). ***F***, Difference in power between remember-first and remember-second tasks in the NB stimulation condition. ***G***, Difference in power between control and NB stimulation in the remember-first task. ***H***, Difference in power between control and NB stimulation in the remember-second task.

**Figure 7.**
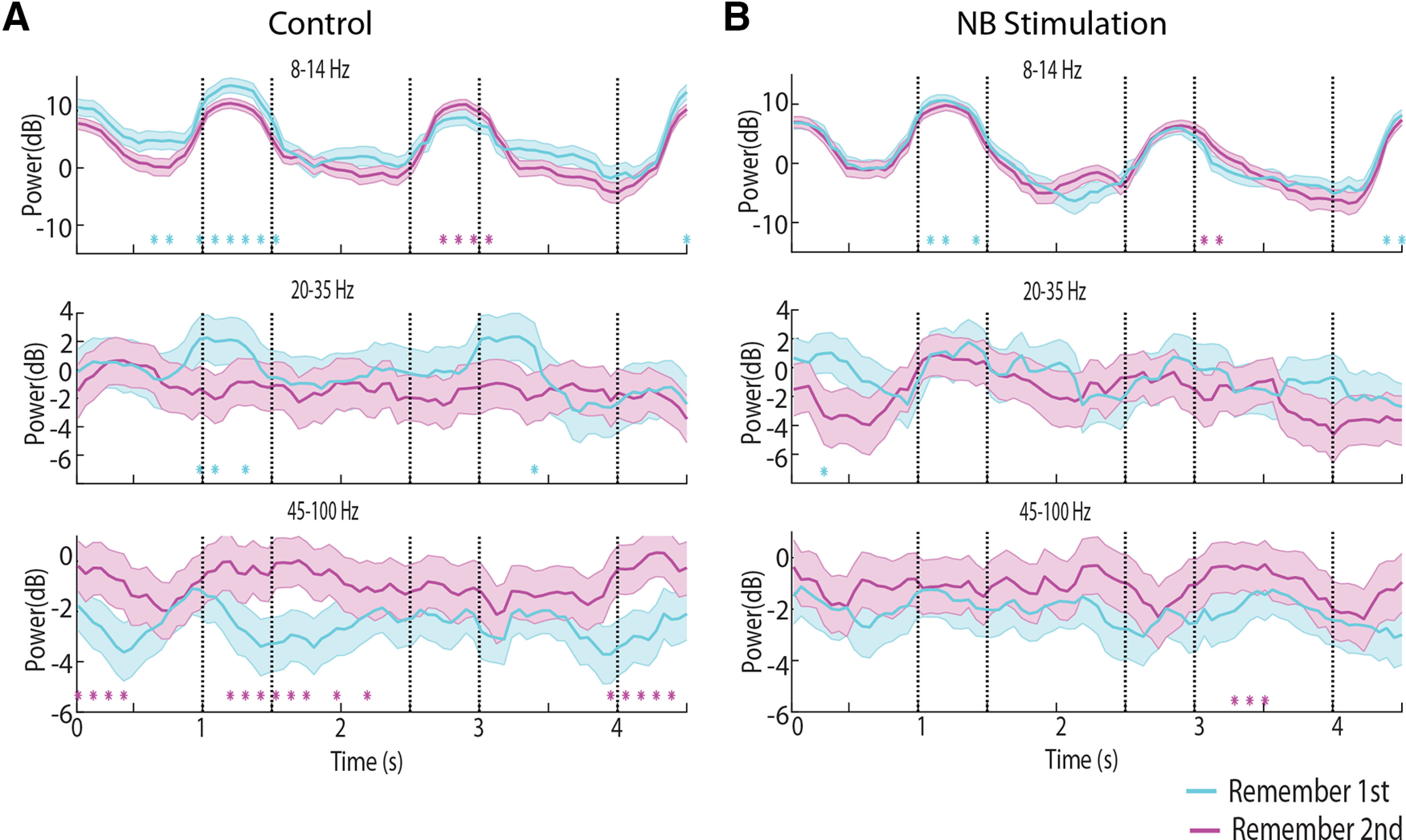
Time course of spectral power for remember first and remember second task. Average power is plotted for the same dataset as that shown in Figure 6. ***A***, Time course of spectral power in the remember-first and remember-second tasks under control conditions. ***B***, Spectral power under NB simulation. Stars represent time points where the difference between the two conditions plotted was found to be significant based on a permutation test, evaluated at the 0.01 significance level.

We next wished to test the effects of NB stimulation in the context of the two variants of the task the monkeys performed, requiring them to remember either the first or second stimulus displayed and how NB stimulation affected each. The remember-second task was generally more difficult for the monkeys, possibly because they learnt the remember-first task earlier (Qi et al., 2021). We wished to test therefore whether γ power, in particular, differed systematically between tasks. This was indeed the case. The spectrogram constructed separately for the two tasks is shown in Figure 6, again averaging all stimulus conditions. In the control condition, power in the γ frequency band was generally higher in the remember-second task compared with the remember-first task (Figs. 6*A*, 7*A*). This difference was maximal and reached statistical significance late in the period of the first stimulus presentation and early in the delay period (Fig. 7*A*).

We also sought to compare power between tasks in the α frequency range. α Power has been implicated in allocation of attention and filtering of distractors (Klimesch, 2012), the timing of which differed in our two tasks. Indeed, we observed that early in the trial, during the fixation period and first stimulus presentation, α power was higher in the remember first than the remember second task (Fig. 7*A*, cyan stars in top panel). This pattern reversed, however, during the second stimulus presentation, which constituted a distractor for the remember-first task but the stimulus to be maintained in memory for the remember-second task (Fig. 7*A*, purple stars in top panel). Using a three-way ANOVA model with epoch, NB stimulation, and task (remember-first/remember-second) factors revealed a significant main effect of task (*F*_(1,70219)_ = 9.79, *p* = 0.002) as well as an epoch × task interaction (*F*_(4,70219)_ = 4.97, *p* = 5 × 10^−4^).

NB stimulation tended to reduce the differences between tasks. Both the elevated γ-band power in the remember-second task, and the alternating pattern of α power during the two stimulus presentations was greatly reduced under NB stimulation (Fig. 7*B*). Only a handful of time points now exhibited a significant difference in the γ band, based on the same permutation test, evaluated at the 0.01 significance level (Fig. 7*B*, lower panel). The overall suppressive main effect of NB stimulation was also highly significant in the three-way ANOVA model (*F*_(1,70219)_ = 72.82, *p* = 1.4 × 10^−17^). In the α band, NB stimulation decreased power in the first stimulus presentation period and increased it in the second, resulting in little difference between tasks. This opposing effect was evident as a significant NB stimulation × task interaction term in the three-way ANOVA model (*F*_(1,70219)_ = 23.6, *p* = 1.2 × 10^−6^).

### Spectral power in error and catch trials

**Figure 8.**
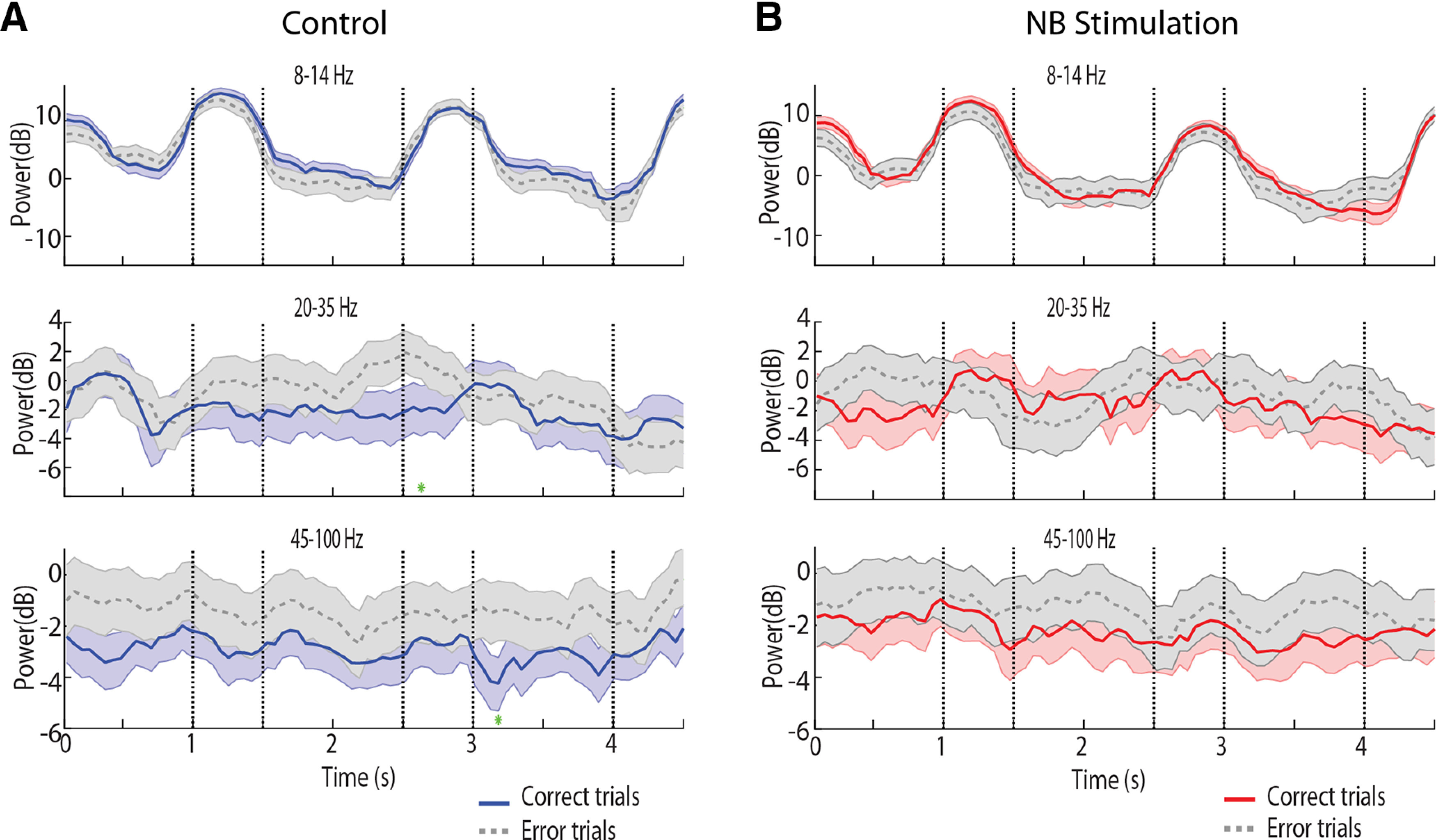
Power spectrum for correct and error trials. ***A***, Time course of spectral power recorded from the prefrontal cortex during the control condition, after subtracting the mean power of the baseline fixation period at each frequency band for correct (*n* = 7846 trials) and error trials (*n* = 1987 trials). ***B***, As in ***A***, under NB stimulation for correct (*n* = 6201 trials) and error trials (*n* = 1561 trials). Stars represent time points where the difference between the two conditions plotted was found to be significant based on a permutation test, evaluated at the 0.01 significance level.

Differences in power between the control and NB stimulation as well as between task conditions do not necessarily imply that these reflect differences in performance of the task. To gain insight on the power distribution that characterized successful task performance, we compared LFP power between correct and error trials (Fig. 8). We averaged responses from all error trials, excluding breaks in fixation, which resulted in premature termination of the trial. The most frequent type of error was a saccade toward the incorrect stimulus (second stimulus in the remember-first task, or first stimulus in the remember-second task).

We first sought to test whether γ power decreased in error trials relative to correct, as would be predicted by theories that consider γ rhythmicity the critical neural variable of working memory maintenance. This did not prove to be the case. If anything, error trials were associated with higher power in the γ frequency range (Fig. 8). When γ power was averaged within each epoch, the effect of correct or error trial outcome was highly significant in a three-way ANOVA model with epoch, NB stimulation, and correct/error factors (*F*_(1,86964)_ = 163.8, *p* = 1.8 × 10^−37^). This difference between correct and error trials was observed for both the control (Fig. 8*A*) and NB stimulation conditions (Fig. 8*B*). Had we relied on the fixation interval as our baseline (as in Fig. 4*C*), this analysis would yield essentially identical γ power in correct and error trials.

A decrease in α power was also evident in error trials (Fig. 8*A*). The main effect of trial outcome was highly significant in the three-way ANOVA model (*F*_(1,86964)_ = 91.3, *p* = 1.3 × 10^−21^). NB stimulation had a similar effect on correct and error trials, evidenced by an absence of a significant interaction term in the model (*F*_(1,86964)_ = 1.78, *p* = 0.18).

**Figure 9.**
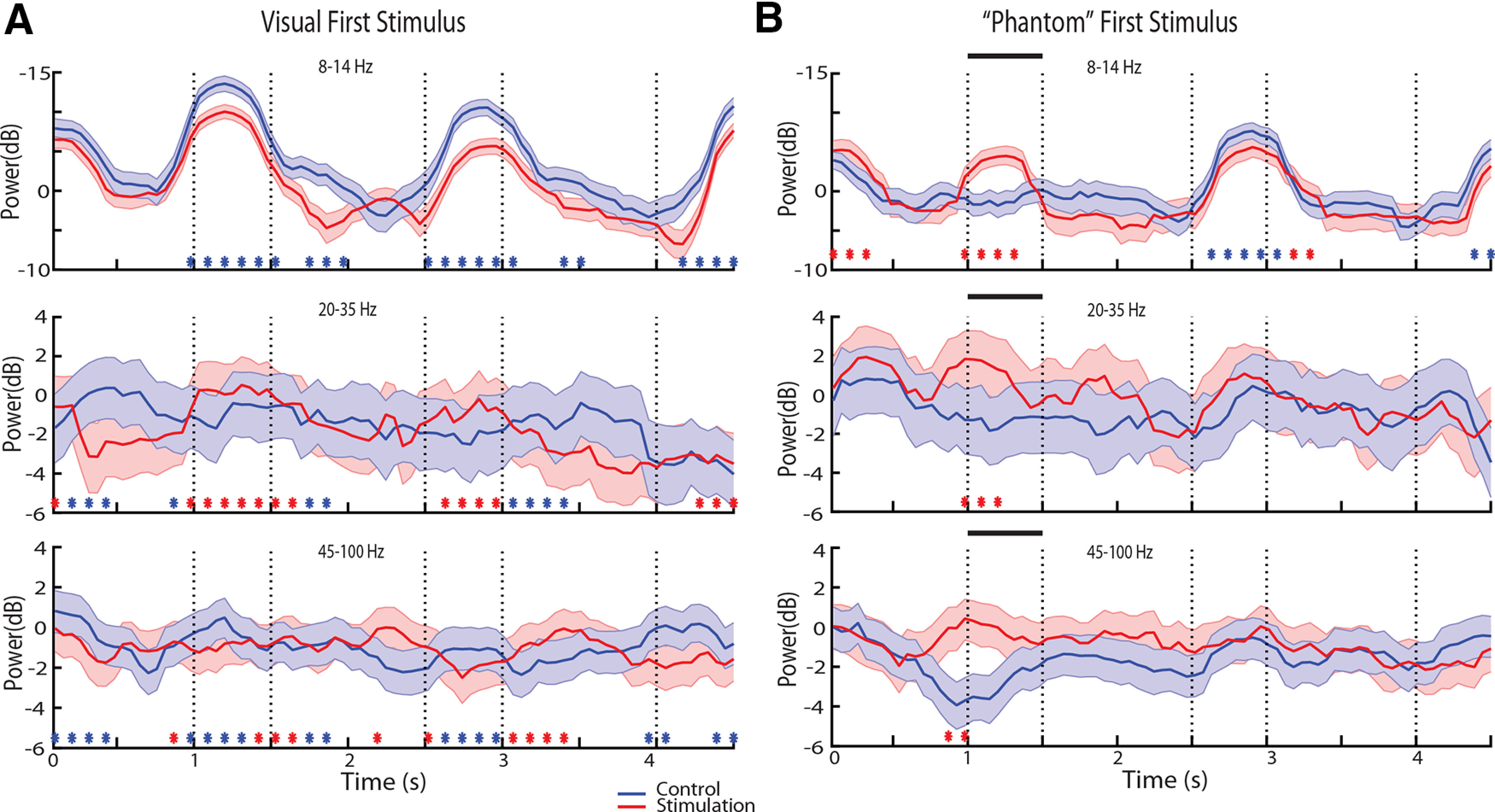
Power spectrum in catch trials. ***A***, Time course of spectral power recorded from the prefrontal cortex during the control condition, after subtracting the mean power of the baseline fixation period at each frequency band for control (*n* = 3334) and NB stimulation (*n* = 2629) trials. ***B***, As in ***A***, for trials in which the first stimulus was omitted (*n* = 884 control and *n* = 613 NB stimulation trials). Horizontal lines represent the expected time of the first stimulus presentation, which was missing in this condition.

We additionally examined spectral power in catch trials, containing no first stimulus. The timing of stimulus presentation was fixed in our task, so that the first stimulus was presented after 1 s of fixation, which presumably the monkeys could time, however, in 10% of the trials the first stimulus was omitted. A second stimulus was still presented at its expected time, in these trials. NB stimulus has been shown to induce “phantom” peaks of firing rate increases during the anticipated time of the missing stimulus presentation (Qi et al., 2021) and we were interested to test whether spectral power would deviate in control and NB stimulation conditions. Indeed, we found an increase in power under NB stimulation, which was evident in all three spectral bands we examined (Fig. 9). Stars in Figure 9 indicate individual time points where the difference statistical significance based on a permutation test evaluated at the 0.01 significance level, indicating most prominently an increase during the cue period. In the α frequency band, despite this transient increase in power under NB stimulation, power quickly receded below the control condition. In the γ frequency band, the result revealed that part of the overall increase in power we observed under NB stimulation (Fig. 3) was associated with this phantom increase.

## Discussion

Our study documents the effects of intermittent NB stimulation on population measures of neuronal activity, as reflected in the LFP. Dysfunction or degeneration of the cholinergic forebrain is responsible for cognitive deficits in humans (Terry and Buccafusco, 2003; Ballinger et al., 2016), an effect that is reliably replicated by animal studies employing cholinergic antagonists or lesions (Chudasama et al., 2004; Croxson et al., 2011; Zhou et al., 2011). NB stimulation has been shown to be effective in improving performance in working memory as well as other cognitive tasks (Blake et al., 2017; Liu et al., 2017, 2018; Qi et al., 2021). As such, it holds promise for the treatment of cognitive decline in conditions such as age-related dementia and Alzheimer’s disease (Subramaniam et al., 2021). However, substantial barriers remain before an effective translation of such an intervention to the clinic (Nazmuddin et al., 2021). Our results provide insights with direct relevance to optimizing the intervention, as they document signatures of rhythmic activity in cases where successful behavioral effects were documented. If similar changes in activity were to be observed in human patients, e.g., through frontal EEG readings, for stimulation sites that provided effective intervention, they could provide an independent means of verifying the effectiveness of the electrode placement, which is a non-trivial problem in clinical targeting (Subramaniam et al., 2021).

### Prefrontal activity mediating improvements in cognitive function

The prefrontal cortex is an area critical for working memory and cognitive plasticity (Constantinidis and Klingberg, 2016; Jaffe and Constantinidis, 2021). It receives innervation from a dedicated sub-region of the NB (Gielow and Záborszky, 2017) and thus is likely to play a role in the cognitive effects of stimulation. The mechanisms through which changes in prefrontal activity mediate improvement in working memory performance are less straightforward. Pharmacological studies suggest that cholinergic agonists increase neuronal activity specifically for the neuron’s preferred stimulus, therefore sharpening its neuronal tuning neurons (Yang et al., 2013; Sun et al., 2017; Dasilva et al., 2019). Muscarinic and nicotinic-α7 inhibitors reduce prefrontal activity and abolishes tuning (Zhou et al., 2011; Yang et al., 2013; Major et al., 2015; Galvin et al., 2020). In contrast to these pharmacological results, NB stimulation revealed general increases in prefrontal activity but no enhancement of a neuron’s preferred stimulus responses, and broadening rather than narrowing of tuning for stimuli (Qi et al., 2021). One possible explanation is that such broadening of prefrontal receptive fields serves to stabilize working memory and make it more resistant to distractors, at least in conditions for which fine discrimination between stimuli is not necessary (Qi et al., 2021).

However, this interpretation is predicated on mechanisms of working memory that depend on attractor models of working memory generation (Compte et al., 2000; Wimmer et al., 2014), a topic that is being hotly debated in the current literature (Constantinidis et al., 2018; Lundqvist et al., 2018). By other accounts, the rhythmicity of discharges is the critical neuronal variable that mediates working memory (Miller et al., 2018). Our current study revealed how NB stimulation altered rhythmicity of LFPs.

### Changes in γ power

We directly tested whether γ frequency power is increased under NB stimulation and whether this could account for improved working memory. LFP γ power is known to be tuned to properties of stimuli held in memory, including the spatial location of the cue in the ODR task (Pesaran et al., 2002; Holmes et al., 2018). In rodents, GABAergic projections from the basal forebrain are electrically coupled and responsible for precisely-timed, rhythmic entrainment of cortical neurons, which is evident in increase of γ-band oscillations when they are selectively activated (McKenna et al., 2013; Kim et al., 2015). NB stimulation (unlike cholinergic pharmacological administration) is likely to activate such ascending GABAergic projections (Walker et al., 1989; Záborszky et al., 2018) and it therefore appeared plausible that NB stimulation might increase γ power of LFPs. Contrary to these predictions, however, our present results show that the aggregate effect of NB stimulation did not increase LFP γ-band power in the prefrontal cortex during the delay periods of the task. An overall increase in γ power was observed after NB stimulation, though this was present throughout the trial rather than the working memory intervals of the task, and its effects manifested themselves in different frequency bands for the two animals. γ Power did exhibit differences between tasks in our experiment, being higher in the more demanding, remember-second task. However, NB stimulation tended to diminish rather than accentuate this difference. Recent results in the rodent auditory cortex did not detect increased γ power after NB stimulation, suggesting that effects of cholinergic stimulation do not depend on γ band increase (Azimi et al., 2020).

An important caveat for the interpretation of our results is the choice of baseline period. We relied on the intertrial interval as the baseline period for most analysis and power from this baseline was subtracted from all trials. Since the color of the fixation point in our experimental design was informative about the rule, we opted to use the inter-trial-interval as the baseline period, rather than the fixation interval. The absence of γ power modulation we report therefore, refers specifically to additional increase in power during the delay period over the baseline, which did not differ between control and NB stimulation conditions. It is also possible that more subtle changes in γ power occur during the maintenance of working memory, for example, short-lived γ-frequency “bursts” (Lundqvist et al., 2016). While we cannot rule out such bust events with the present analysis, their cumulative effect in the delay period was not visible across trials.

### Changes in α power

Although it was not our a priori hypothesis, changes in the α band power proved the most sensitive indicator of different conditions in our experiment. α Power decreased by NB stimulation compared with control, particularly in the delay periods of the task, when the subjects need to maintain the relevant information in memory. α Power also differentiated the remember-first and remember-second tasks, being higher during the stimulus presentation period of whichever stimulus were to be maintained in memory. Similarly, α power was reduced in error trials compared with correct ones. Finally, NB stimulation produced a phantom increase in α power in periods over which a stimulus appearance was expected but did not occur.

α Power in EEG studies has been traditionally viewed as indicative of an idling state of cortex and elevated α power predictive of errors in processing because of lack of engagement in a task; however, more recently this notion has been shifted toward of a view that α power in sensory cortex reflects active suppression of task-irrelevant information (Klimesch, 2012; Clayton et al., 2015; Peylo et al., 2021). For example, when attention is shifted to color as opposed to motion in the context of a task, or to one hemifield over another, or to sensory modalities other than vision, α power increases in the cortical areas that are irrelevant for the task: the dorsal visual stream, ipsilateral hemisphere, or the visual cortex, respectively (Foxe et al., 1998; Snyder and Foxe, 2010; Doesburg et al., 2016). Increased entraining of α power by means of TMS rhythmic stimulation can bias visual perception in the ipsilateral visual field (Kasten et al., 2020). In the context of working memory, increased α power in posterior EEG electrodes is observed in tasks requiring suppression of distractors, and with increasing memory load (Hu et al., 2019).

Such a role of α band power is less clear in the frontal cortex, however. Reversed patterns of α rhythmicity between sensory and association cortex have been described in neurophysiological experiments in monkeys (Bollimunta et al., 2008). In the ODR task, α power is known to be elevated during the stimulus presentation, but does not otherwise appear tuned to stimulus properties in the delay period (Holmes et al., 2018). In our experiment, α power was indeed higher during the presentation of a stimulus that needed to be remembered (the first one in the remember-first task, and the second one in the remember-second task) and higher in correct than error trials. In this context, the decrease of α power by NB stimulation during the delay period would suggest that network is less likely to shift attention to the second stimulus. This interpretation is broadly in agreement with the analysis of behavioral and neurophysiological results (Qi et al., 2021): NB stimulation tended to stabilize the activity of prefrontal neurons and make memory of the stimulus less likely to shift (except in the case of nearby stimuli). Our current results suggest that these changes in attentional selection were more likely to be reflected in the α rather than the γ frequency power.

## Synthesis

Reviewing Editor: Mark Laubach, American University Decisions are customarily a result of the Reviewing Editor and the peer reviewers coming together and discussing their recommendations until a consensus is reached. When revisions are invited, a fact-based synthesis statement explaining their decision and outlining what is needed to prepare a revision will be listed below. The following reviewer(s) agreed to reveal their identity: Charles Wilson.

Two experts reviewed your manuscript and raised some issues that you should address before the manuscript can be considered for publication. The raw reviews are given below. Please revise your manuscript to address all points that were raised. Thank you.

Reviewer #1

This manuscript describes the effects of intermittent stimulation of the nucleus basalis on the frequency bands of the local field potential response in macaque prefrontal cortex during the performance of oculomotor working memory paradigms. Previously, other studies have shown that intermittent, but not continuous stimulation, augments subject performance in these tasks. Additionally, electrophysiological studies have shown that intermittent NB stimulation can augment the activity of prefrontal neurons, but thereby broadening the tuning width of stimulus selectivity. Those studies have posited that the broadening of tuning width stabilizes mnemonic representations in trade-off with accuracy of those representations and thereby leads to improvement in behavioral performance when distractors are clearly separated from targets, but disruptive to performance when the distractors are spatially close to the targets.

While those studies have mainly focused on spiking activity in prefrontal cortex, less is known about the effects of NB stimulation on the field potential signatures of task performance in prefrontal cortex. Here, the authors find that intermittent NB stimulation decreases alpha band activity in prefrontal cortex, while having less prominent effects on gamma band activity. Further, they find that alpha band augmentation in the stimulus period depends on the task context of the stimulus (whether it is a target or distracter). The results regarding gamma activity indicate that substantive modulation of gamma band activity need not accompany improvements in behavioral performance, an observation that is of interest given the recent studies describing working memory processing in the context of gamma bursts.

1) The authors mention in the methods that while subjects were trained with eight stimulus locations, only two were employed during the recording. In the results they state the two stimuli were selected in a randomized order from the eight stimulus locations. To clarify, the stimulus locations were not selected to align with the response fields of neurons that may have been concomitantly recorded from the same electrode?

2) How many tungsten electrodes were introduced in a given session on average?

3) What kind of electrodes were employed for the deep brain stimulation and what were their impedances?

4) In the methods, the authors state that “Stimulation was delivered with biphasic, negative first, unipolar 200 μA pulses with 100 μs per phase, and 80 Hz pulses were delivered for 100 ms.” Later, in the description of the blocks of stimulation, it is stated that stimulation carried out for 15s followed by 45s of no stimulation. So, the stimulation during the 15s was 100ms for each second?

5) The authors describe in the methods that stimulation was applied in a block paradigm with trials with and without stimulation collected in the same session. The blocks of trials consisted of 60 trials with 15s interludes of stimulation. This is shown in Figures 1D and 1E. Were the blocks of no stimulation (Figure 1E) interspersed with the blocks of stimulation (Figure 1D) on any given day? If so, typically how many blocks of stimulation and no stimulation occurred in a given experimental session?

6) The manuscript states: “The baseline period was defined to be last 1 second in the inter-trial interval apart from the trials in which the NB stimulation was delivered"

It is not clearly mentioned but based on the description of the task, the ITI can be inferred to be ∼ 5s in this paradigm. During the intertrial interval, presumably the monkeys were free to move their eyes and thus their gaze position would be random from ITI to ITI. Could this possibly affect the baseline normalization in a nontrivial way?

7) The authors mentioned that two additional trial types, where either stimulus 1 was omitted on Remember 2nd trials or stimulus 2 was omitted on Remember 1st trials. Were these included in the analyses in Figures 3 and 4?

8) In Figure 5, the authors describe analysis of correct and error trials. They state in the results that gamma power was higher on error trials than correct trials. However, this effect only seems palpable at one time point in the figure. Is this difference significant in the trial-average analysis that the authors described in the results pertaining to data in Figure 2?

9) How were the error trials defined for the purposes of Figure 5. In Remember 1st trials, a salient error would be to go for the second stimulus location instead. Similarly, in Remember 2nd trials a salient error would be to go for the 1st stimulus. Additionally, there can be errors where the animal makes a saccade to an unrelated location or does not respond at all. Were these analyses restricted to the former error types?

10) Qi et al (2021) described behavioral effects of similar NB stimulation, finding that even though neuronal tuning was broadened, task performance went up, except when the second stimulus was spatially close to the first stimulus, in which case the behavioral performance was worse for the Remember 1st condition, but not the Remember 2nd. This indicates a distractor effect of the nearby second stimulus. Here, the authors argue in the discussion that decreases in alpha power observed are more likely to reflect the distracter resistance augmentation due to NB stimulation. Do the authors see differences in the alpha power before and after stimulation in Remember 1st trials when the second stimulus was spatially close or contralateral to the 1st stimulus that might support this hypothesis?

11) Qi et al (2021) had described an intriguing finding of anticipatory “stimulus-like” activity in these stimulus-omitted trials presumably due to the monkey’s expectation based on experience that a stimulus would be presented. Did the frequency band power changes described also occur during this anticipatory activity? Is there enough statistical power in terms of trials to study this?

12) Typo on last paragraph page 10: “Averaging power across each task epoch revealed no significant difference between power in the control and NB-stimulation condition in the beta frequency band”. This should be gamma frequency band here.

Reviewer #2

This study investigates cholinergic neuromodulation through the stimulation of the NB in macaques. The authors sought to understand how NB stimulation affects global measures of neural activity, specifically LFPs, during working memory tasks. They found differences between tasks in gamma power (higher in the more demanding task), but NB stimulation tended to diminish rather than accentuate this difference. They also found that alpha power was higher during the presentation of a stimulus that needed to be remembered and that NB stimulation generated a decrease of alpha power during the delay period. They conclude that NB stimulation tends to stabilize the activity of prefrontal neurons.

The manuscript is clearly written. The data are valuable to the community and should be published. We mainly have comments on the analysis, which could be improved to bring out or rule out certain potential effects, and to strengthen our understanding of the statistical relationships.

1. Whilst the studies in intact monkeys do indeed suggest some promise for the clinical application of NB stimulation in patients, the introduction and discussion are a little unbalanced on this point - as the authors will be aware there remain plenty of doubts about the application of this approach in a clinical situation, notably outside of the brain-intact monkey model (see e.g. Nazmuddin et al 2021 Sci Rep). In addition, the cholinergic lesion literature has a major bearing on this field but remains uncited (for example Chudasama et al Learning & Memory 11, 2004; Croxson et al Nat Neurosci 14, 2011).

2. The authors use trial-by-trial baselining of LFP signal, but the block design of the task means individual trials are not independent. The first and subsequent trials of the block may well have pre-trial activity that reflects different cognitive expectations of the animals. Trial-by-trial baselining of LFP signal (last 1s in the ITI) might potentially mask cognitive effects - can the authors show that the baseline is stable for LFP measures between trials and between blocks?

3. Frequency bands significantly vary between individuals. Here fixed bands were used (p8). Can the authors demonstrate that the bands used here reflect identified peaks of activity in the two monkeys individually? Because this is necessary, for example, to collapse data across the two animals. The analysis approach might be masking important effects if the frequency bands differ between individuals. For example this might explain monkey differences in the beta range that we see in figs 2E and 2F. Inspection of individual power spectra, or even better Fooof-ing the data (Donoghue et al Nat Neurosci 23, 2020) would suffice to check whether this is the case.

4. Much of the subsequent statistical analysis relies on collapsing data into these frequency bands. Beyond the idea that they may vary between individuals, it might also be preferable to perform the statistical analysis across the whole time-frequency space. The cluster-based permutation approach described by Maris & Oostenveld (J Neurosci Methods 164, 2007) is one example where the statistical effects can be studied without recourse to imposed frequency bands. For example, the key effect reported is a modulation in the alpha band (Fig 2D. But inspection of Fig 2C suggests that this effect is present roughly 0-10Hz with little temporal resolution, a rather different result to the one reported.

5. Further on the frequency analysis, the authors cite the work of Lundqvist, yet do not account for a major element of these studies, which is that the activity they report occurs in very discrete time-resolved bursts, not as a continuous oscillation. Several labs have demonstrated that considering this temporal feature of the oscillatory signal is important for interpreting it. So as with the frequency bands, ignoring it might mask important effects (of course, it might also not). I think the authors should address this explicitly - ideally through analysis.

6. The authors describe several contrasts that produce varying effects on the oscillatory power. It would be helpful to have some simple statistical modelling so that these effects can be considered together. For example, in Fig 4 there are stimulation and task effects, a statistical approach allowing us to compare them would be welcome.

7. “Electrode placement was guided by MR imaging” (p9) yet we have virtually no information about recording locations beyond a large blob on the image of Fig 1A. Please can we have more detailed information on recording locations?

Typos & minor points:

- last sentence of the abstract - “an” not “and", perhaps?

- methods: The age of the monkeys should be included

- significance marker referred to as both asterisks and stars for Fig 2D (top p10)

- bottom p14 - “subjects"

- it would be helpful if the Correct/Error colours were consistent in Fig5 between A and B.
